# Self-Inserted Knotted Wire Through the Urethra: A Case Report

**DOI:** 10.7759/cureus.70628

**Published:** 2024-10-01

**Authors:** Hasan R Al Shabaan, Hussain M Almodhi, Abdulelah A Alabdulwahab, Mohammed T Aldoukhi, Hasan Y Alkhalifa

**Affiliations:** 1 Urology, King Fahad Hospital Hofuf, Al Ahsa, SAU; 2 Emergency Department, King Fahad Hospital Hofuf, Al Ahsa, SAU; 3 Urology, Almoosa Health Group, Al Ahsa, SAU

**Keywords:** dysuria, endourology, foreign body, hematuria, urethra

## Abstract

Foreign bodies in the lower urinary system are relatively uncommon. The foreign body can cause significant discomfort, hematuria, or infection of the urinary system. Diagnosis is usually made by clinical examination and pelvic X-ray. The case we present is interesting in that the foreign body, that is the wire, coiled and made a knot within the bladder. We report a case of a 25-year-old male who presented with a history of dysuria after the insertion of a foreign body into the urethra. Examination showed a wire protruding from the urethra with no bleeding or discharge. X-ray showed a wire from the meatus, coiled and tied in the bladder. Open cystostomy and extraction of the wire were done successfully. Foreign bodies are usually a result of psychological illness. Thereby, psychiatric evaluation is needed in such a presentation. Management should be started by a trial of retrieval or, if failed, endoscopically or by open cystostomy.

## Introduction

Although foreign bodies in the lower urinary system are uncommon, they have been recorded in a few cases. Several factors may lead to the insertion of foreign bodies, for example, sexual curiosity, autoerotic stimulation, or being intoxicated during sexual practice [1,2]. The age of presentation usually ranges from 15 to 55 years [3]. The material of foreign bodies has been made of a variety of items, including wire, screws, and ballpoint pens, as well as some animals or animal parts such as snakes, leeches, or bones [2,4,5]. The foreign body can be left in place for a long period of time with little discomfort. However, in most cases, the foreign body causes significant discomfort, hematuria, dysuria, or infection of the urinary system [6].

Herein, we present an interesting case of a patient who inserted a wire into the urethra up to the bladder with a spontaneous knot that formed in the bladder.

## Case presentation

**Figure 1:**
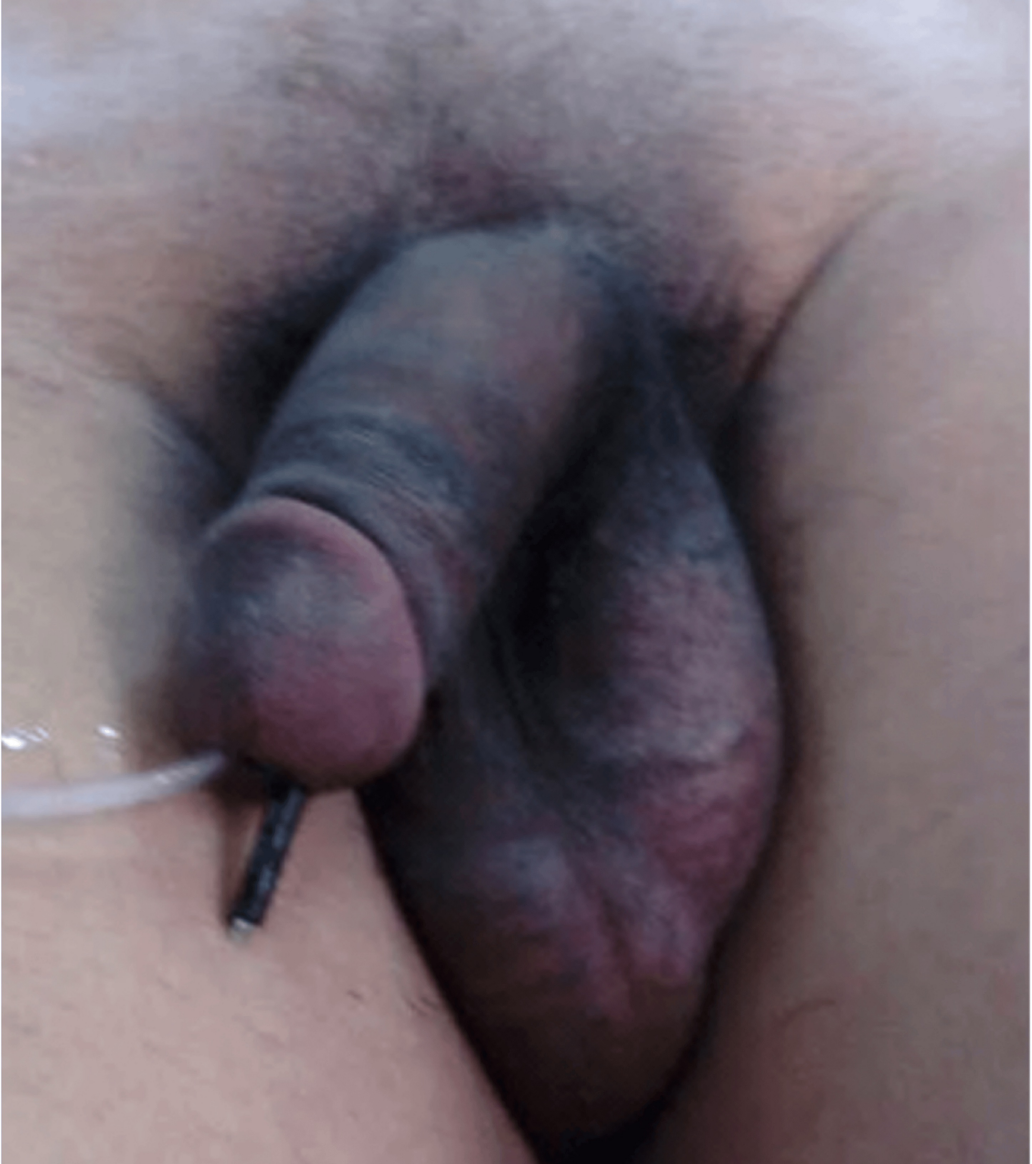
The foreign object is seen protruding from the urethra Upon examination, the patient had a foreign body protruding from the urethra with no signs of infection.

**Figure 2:**
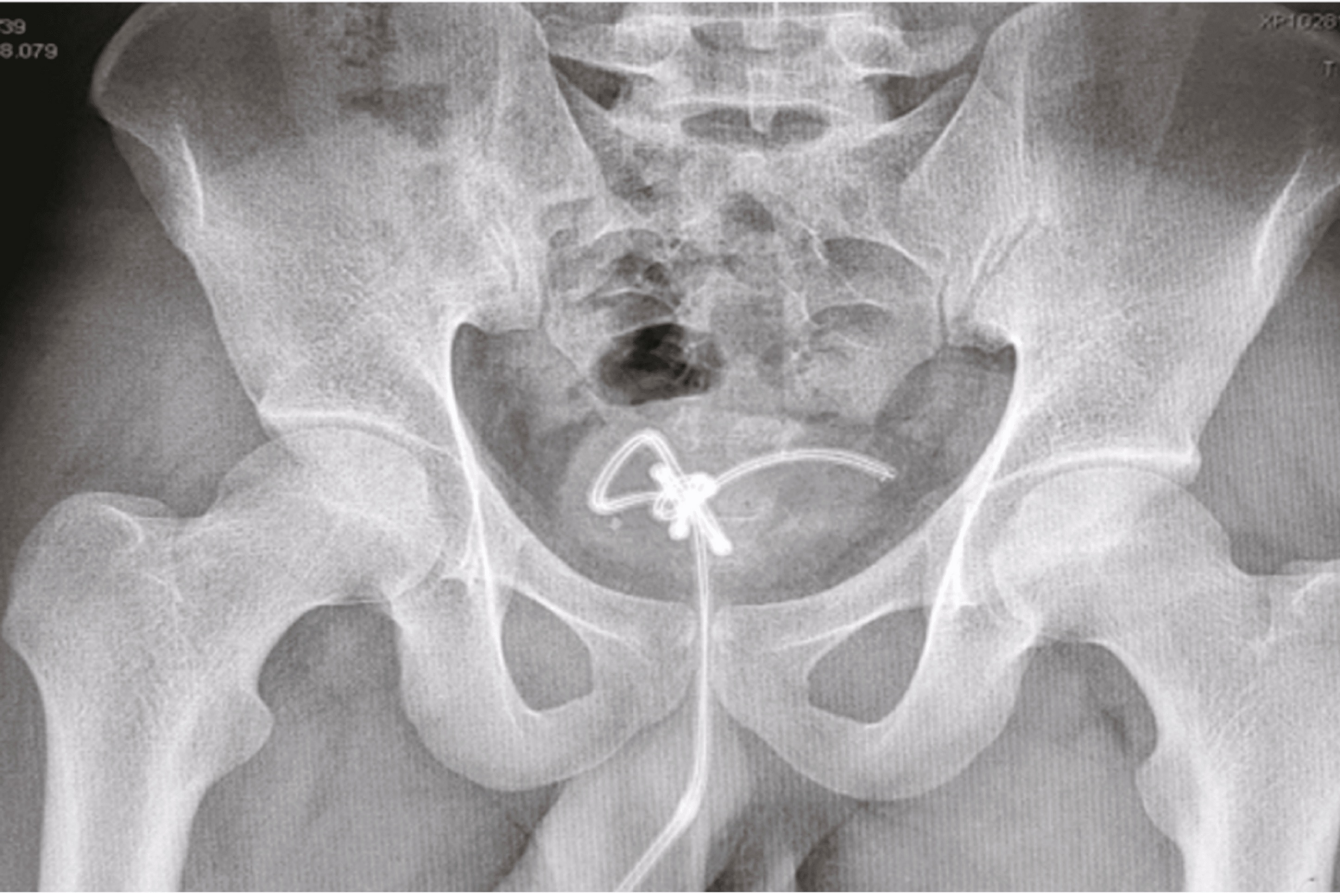
Pelvis X-ray of the foreign body Pelvis X-ray showing the foreign body (wire) knotted inside the bladder.

**Figure 3:**
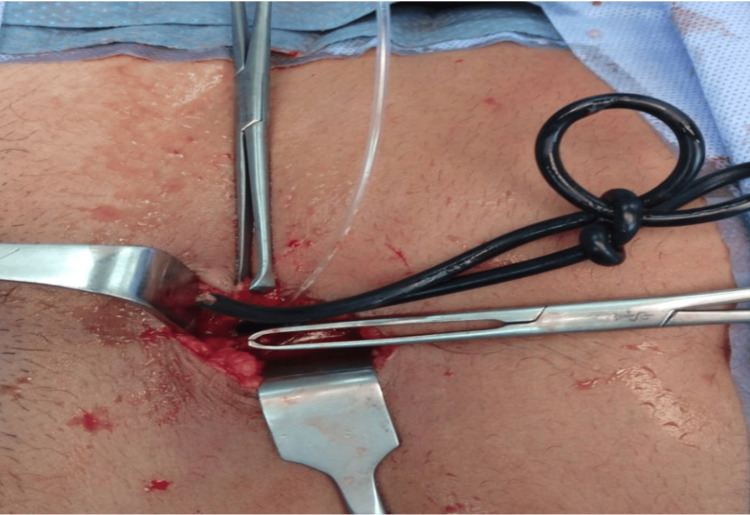
Intraoperative photo of the foreign body Intraoperatively, the wire was seen knotted in the bladder.

A 25-year-old male patient presented to our emergency department with a history of dysuria following the self-insertion of a foreign body into the urethra for more than one-day duration. He denied any history of hematuria, abdominal pain, or drug abuse. On examination, a visible wire was seen projecting from the external urethral meatus with no bleeding or urethral discharge (Figure 1). A trial of gentle retrieval was done, which was unsuccessful. X-ray abdomen and pelvis showed a long wire from the external urethral meatus coiled and tied in the urinary bladder (Figure 2). The decision was made to prepare the patient and take him to the operating room. Open cystostomy was performed with extraction of the wire, showing that the wire was knotted spontaneously in the bladder (Figure 3). A drain and a Foley’s catheter were inserted. Post-operatively, the patient was doing well with minimal output from the drain. On the third day post-op, the drain was removed, and the patient was discharged home. On follow-up, the patient was seen in good condition with no symptoms.

## Discussion

The number of self-insertions of foreign bodies into the urethra and bladder has increased significantly over the last decade, especially among male individuals [5]. This could be related to the high incidence of psychiatric disorders [7]. The clinical presentation of insertion of a foreign body into the urethra ranges from asymptomatic to mild lower abdomen or penile pain, dysuria, hematuria, bleeding per urethra, pyuria, swelling at the glans or shaft of the penis, frequency, retention, or fever [8]. Usually, the presentation is delayed due to a feeling of embarrassment and is frequently associated with urethral injury or migration of the foreign body due to various trials of self-removal of the object [5,8]. Foreign body insertions into the lower urinary tract have a low incidence in general, with males 1.7 times more likely to commit the act than females [3,5,8].

The most common motive for such kind of behavior is sexual or erotic stimulation, in the form of masturbation or sexual gratification [2,5,6]. Other motives include psychiatric illness, drug intoxication, or sexual curiosity [5,6,9].

Diagnosis is frequently made by history and physical examination. Radiography by x-ray pelvis, intravenous urogram, cystogram, ultrasound, or computed tomography (CT) is necessary to identify the size, location, and number of foreign bodies inserted [1,5,8].

Definitive management for these patients is to remove the foreign body with minimal complications such as urethral or bladder injury, urinary tract infections, or peritonitis [5,6].

Different methods are available for the removal of the foreign body and should be selected according to the size, shape, and location of the foreign body. These procedures consist of meatotomy, cystoscopy, urethrotomy, and open cystostomy. In cases where the endoscopic procedures are not successful, open surgical removal is necessary. Objects in the penile urethra can be removed by urethroscopy or open extraction. For intravesical objects, cystoscopy or open cystostomy is recommended for removal [5,10].

The optimal way to remove a foreign body should be chosen after considering the size and nature of the object, any related urinary tract injuries, and the patient's condition.

## Conclusions

Foreign bodies in the urethra are uncommon. It is usually a result of a psychological illness leading to this kind of behavior. Hence, all patients with such a presentation should be referred for psychiatric evaluation. A trial of retrieval should be done initially, if failed, cystourethroscopy or open cystostomy, as was done in our case, is needed to remove the foreign body. 
